# Repetitive bout of controlled soccer heading does not alter heart rate variability metrics: A preliminary investigation

**DOI:** 10.3389/fneur.2022.980938

**Published:** 2022-11-25

**Authors:** Jonathan David Smirl, Dakota Peacock, Joel Stephen Burma, Alexander D. Wright, Kevin J. Bouliane, Jill Dierijck, Paul van Donkelaar

**Affiliations:** ^1^Concussion Research Lab, University of British Columbia, Kelowna, BC, Canada; ^2^Faculty of Kinesiology, University of Calgary, Calgary, AB, Canada; ^3^Sport Injury Prevention Research Centre, University of Calgary, Calgary, AB, Canada; ^4^Human Performance Laboratory, University of Calgary, Calgary, AB, Canada; ^5^Faculty of Medicine, Hotchkiss Brain Institute, University of Calgary, Calgary, AB, Canada; ^6^Integrated Concussion Research Program, University of Calgary, Calgary, AB, Canada; ^7^Alberta Children's Hospital Research Institute, University of Calgary, Calgary, AB, Canada; ^8^Libin Cardiovascular Institute, University of Calgary, Calgary, AB, Canada; ^9^Southern Medical Program, University of British Columbia, Kelowna, BC, Canada; ^10^Division of Neurology, Department of Pediatrics, BC Children's Hospital, Vancouver, BC, Canada; ^11^University of British Columbia, Vancouver, BC, Canada; ^12^Experimental Medicine, University of British Columbia, Vancouver, BC, Canada; ^13^School of Physiotherapy, Faculty of Health, Dalhousie University, Halifax, NS, Canada

**Keywords:** repetitive football/soccer heading, heart rate variability, autonomic function, sub-concussive impacts, sport concussion assessment tool 3, SCAT3

## Abstract

**Objectives:**

There is elevated unease regarding how repetitive head impacts, such as those associated with soccer heading, contribute to alterations in brain function. This study examined the extent heart rate variability (HRV) and cardiac baroreceptor sensitivity (BRS) metrics are altered immediately following an acute bout of soccer heading.

**Methods:**

Seven male elite soccer players (24.1 ± 1.5 years) completed 40 successful soccer headers in 20-min. The headers were performed under controlled circumstances using a soccer ball launcher located 25 meters away and using an initial ball velocity of 77.5 ± 3.7 km/h (heading condition). An accelerometer (xPatch) on the right mastoid process quantified linear/rotational head accelerations. Participants also completed sham (body contact) and control (non-contact) sessions. A three-lead ECG and finger photoplethysmography characterized short-term spontaneous HRV/cardiac BRS, before and after each condition. The SCAT3 indexed symptom scores pre-post exposures to all three conditions.

**Results:**

During the heading condition, cumulative linear and rotational accelerations experienced were 1,574 ± 97.9 g and 313,761 ± 23,966 rad/s^2^, respectively. Heart rate trended toward an increase from pre- to post-heading (*p* = *0.063*), however HRV metrics in the time-domain (*ps* > *0.260*) and frequency-domain (*ps* > *0.327*) as well as cardiac BRS (*ps* > *0.144*) were not significantly changed following all three conditions. Following the heading condition, SCAT3 symptom severity increased (*p* = *0.030*) with a trend for symptom score augmentation (*p* = *0.078*) compared to control and sham.

**Conclusion:**

Whereas, symptoms as measured by the SCAT3 were induced following an acute bout of controlled soccer heading, these preliminary findings indicate they were not accompanied by alterations to autonomic function. Ultimately, this demonstrates further research is needed to understand the physiological underpinnings of alterations in brain function occurring immediately after a bout of soccer heading and how these may, over time, contribute to long-term neurological impairments.

## Introduction

Around the globe, more than 265 million individuals partake in soccer making it the world's most prominent sport (1) and one that also results in substantial risk for head injuries to occur (2–4). This is very likely at least partially due to the unique aspect of this sport in which players use their heads to contact the ball and direct it during play. Previous research has demonstrated soccer heading occurs ~4 to 10 times per game with variations dependent upon age, sex, and player position [e.g., (5)]. Beyond the immediate effects of soccer heading on brain function, there is emerging concern that the number of repetitive sub-concussive impacts a soccer player has over their career may be associated with the development of long-term neurodegenerative disorders (6–9). Nevertheless, there is currently a paucity of data providing insight into the magnitude and total exposure necessary to cause alterations in brain function in the immediate aftermath of a bout of soccer heading.

The autonomic nervous system functions to maintain homeostasis through the unconscious regulation of various bodily functions (e.g., heart rate, respiration, digestion, etc.) (10). Previous research has demonstrated autonomic function can be aberrant in certain pathophysiological states [e.g., Parkinson disease (11), multiple sclerosis (12), traumatic brain injury (13–15), etc.], through heart rate variability (HRV) and baroreceptor sensitivity (BRS) domains. HRV is a technique designed to examine the activity of the autonomic nervous system through the variations in consecutive R-R intervals (16). Conversely, BRS maintains cardiovascular homeostasis through a negative feedback loop by balancing input from the sympathetic and parasympathetic nervous systems during acute alterations in blood pressure (17).

Autonomic function has been found to be dysregulated following concussion (13–15, 18), though the exact physiological underpinning of this phenomenon remains unknown. Furthermore, there is a relative void within the current literature examining the effects repetitive heading impacts has on autonomic function. To our knowledge there has only been one study which investigated an acute bout of five soccer head impacts and found minimal alterations in HRV parameters (19). As such, there is a substantial lack of understanding regarding the physiological changes that occur to the autonomic nervous system from both concussive and sub-concussive head impacts. Therefore, this investigation sought to examine the acute effects a controlled bout of soccer heading using a soccer ball launcher has on the autonomic nervous system relative to sham (body contact) and control (non-contact) sessions. It was hypothesized repeated sub-concussive impacts in the form of soccer heading would lead to an elevated sympathetic response in HRV and cardiac BRS metrics that would not be present during sham (non-head impacts) and control (no impacts) conditions.

## Materials and methods

### Participants

Seven male soccer players (age: 24.1 ± 1.5 years; BMI: 25.5 ± 1.6 kg/m^2^) participated in this investigation, which used a crossover design that has also examined cerebral autoregulation (20), neurovascular coupling (21), and blood-based biomarkers (22) within the same three randomized conditions (heading, sham, and control). All individuals had a minimum of 5 years of soccer playing experience and refrained from caffeine, alcoholic beverages, smoking, and exercise for 12 h prior to testing. Participants were healthy with no history of cardiac, respiratory, neurological, vascular, or severe neurodevelopmental disorders. Testing procedures were explained prior to data collection to ensure all participants were familiar with them. Written informed consent was obtained and ethical approval was through the University of British Columbia clinical research ethics board (H14-00368). Participants were compensated $50 CAD for each testing session, for a total compensation of $150 CAD across the duration of the study.

### Study design

On each testing day, spontaneous short-term HRV and cardiac BRS was quantified using a pre-test, exposure, post-test design through electrocardiography and finger plethysmography (23, 24). These variables were measured while each individual quietly stood for 5 min, before and roughly 15–20 min after each condition. Participants completed these conditions with an average of 26.1 ± 25.2 days between testing conditions in a pseudo-random order, in brief the conditions consisted of:

i) Heading—participants performed 40 headers in 20 min with ~30 s between each trial. They stood ~25 m from a JUGS machine (JUGS International, Taulatin, Oregon, USA). In the case of an unsuccessful trial, a second soccer ball was launched within the 30 s time frame. The soccer balls used were FIFA regulation size 5 ball inflated to 13 psi and propelled from the JUGS machine at an average of 77.5 ± 3.7 km/h, which was recorded with a Bushnell Velocity Speed Gun (Bushnell Outdoor Products, Richmond Hill, Ontario, Canada).
ii) Sham—Participants performed 40 ball contacts in 20 min with any part of the body other than the head. Other than this requirement, all the other details of this condition were the same as the Heading condition. This intervention was performed to determine if alterations to the autonomic nervous system require head contract or if they could arise from body contact or “*whiplash-like*” effects (25).
iii) Control—No soccer balls were launched in this condition, as participants were taken to the testing area, and completed 20 min of quiet rest, before returning to the laboratory for post-condition data collections.

For a greater description of the heading, sham, and control protocols the reader is directed to (21).

### Lab-based instrumentation

A three-lead electrocardiogram (ECG) and finger photoplethysmography, with a brachial cuff to adjust finger and brachial artery height differences (Finometer; Finapres Medical Systems, Amsterdam, The Netherlands), was attached to each individual before and following the three aforementioned conditions (26, 27). All data were sampled at 1,000 Hz (PowerLab 8/30 ML880; AD Instruments) and stored for offline analysis using commercially available software (LabChart version 7.1; AD Instruments). All measurements were collected at the same time of day to control for diurnal variation (28, 29).

Consistent with other research in the soccer heading field, linear and angular acceleration were measured using the xPatch (X2 Biosystems; Seattle, WA) system placed over the right mastoid process of participants during both the header and sham conditions (20–22, 25, 30–32). These sensors monitor three axes of translational acceleration as well as three axes of angular velocity with a 1,000 Hz sampling frequency. Head impact accelerations exceeding the threshold of 10 g were automatically recorded by the device and the associated peak linear/rotation accelerations (PLA, PRA) as well as the average impact duration were quantified. In the event, the impact from heading the soccer ball (or contact to the body in the sham condition) did not meet or exceed the standard 10 g threshold of the device, the impact was then coded as 0 g for interpretations of average/cumulative impact exposure levels. For this paper, we analyzed average and total linear and rotational acceleration in each condition. Others have shown soccer heading effects based on number of head impacts (33, 34) or time between impacts (35), however, because these variables were held relatively constant in the current study, we felt that average and cumulative impact magnitude variables would capture head impact exposure most appropriately.

The total number of symptoms and symptom severity scores were recorded with the third edition of the Sport Concussion Assessment Tool (SCAT3) before and after the three interventions (36). This tool contains a Likert scale ranging from 0 (no symptom) to 6 (severe) with 22 symptoms regarding somatic, cognitive, and neurobehavioral functions. The total number of symptoms (range: 0–22) and symptom severity were calculated by totaling the severity for each symptom (range: 0–132). Participants had a follow up call in the evening following soccer heading to assess symptom persistence. No individuals reported any lingering symptomology at this time point.

### Data processing

Following at least 1 min of standing, short-term HRV/cardiac BRS measures were collected through 5 min of quiet standing in accordance with established guidelines (37, 38) using commercially available software (Version 1.0, Ensemble, Elucimed, Wellington, NZ). The outcome variables for HRV included the root mean square of successive normal sinus QRS complexes interval (R-R interval) differences (RMSSD), number of successive R-R intervals that differ by more than 50 ms (NN50), the percentage of R-R intervals that differ by more than 50 ms (pNN50), and low frequency (LF) and high frequency (HF) power (23, 37–40). The cardiac BRS was quantified through the LF gain (0.07–0.20 Hz) metric (23, 24, 41).

### Statistical analysis

Statistical analyses were conducted with SPSS v.25.0 (IBM Crop, Armonk, NY). A three (condition: heading, sham, control) by two (time: pre, post) repeated measures analysis of variance was performed. Bonferroni *post-hoc* analyses were conducted to determine significant condition effects, with *a priori* Bonferroni corrected simple effects comparisons for condition or time. Data are presented as mean ± standard deviation. Significance was set *a priori* at *p* < 0.05.

## Results

### xPatch impact sensor

Greater than 98% of the headers were recorded by the xPatch Sensor as above the threshold of 10 g, with an average of 40.7 ± 3.6 g and 8097.8 ± 807.3 rad/s^2^ of linear and rotational acceleration, respectively. This translates to 1574.7 ± 97.9 g of total linear acceleration and 313760.6 ± 23966.4 rad/s^2^ of total rotational acceleration during the soccer heading intervention. Conversely, during the sham condition, <1% of the body contacts registered an impact >10 g, with an average linear acceleration of 3.2 ± 5.7 g and rotational acceleration of 827.9 ± 1424.5 rad/s^2^ (these values are underestimates as any impact below 10 g was registered as 0 g). Thus, the cumulative exposure during the sham condition was 1574.7 ± 97.9 g and 1284.8 ± 2453.2 rad/s^2^, for linear and rotational acceleration, respectively.

### Sport concussion assessment test – 3rd edition (SCAT3)

**Figure 1:**
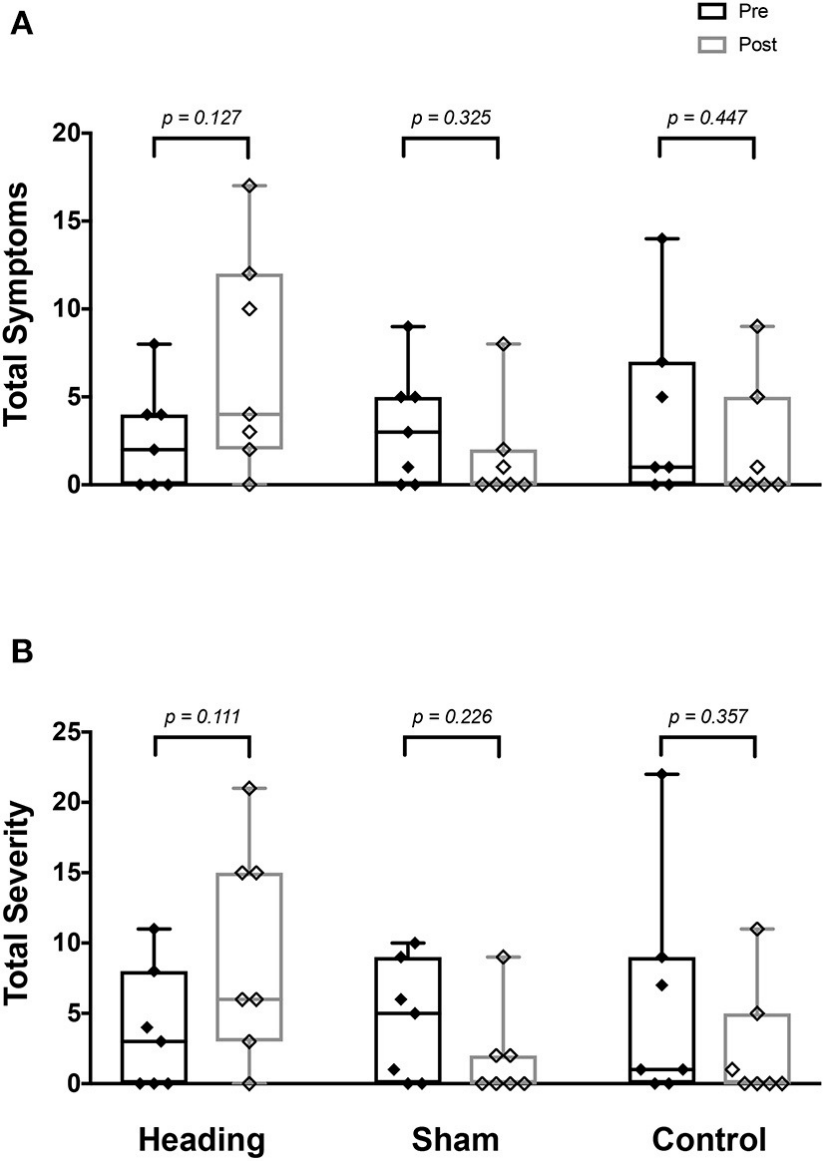
SCAT3 symptom metrics measured before and after exposure to heading, sham, and control conditions across all participants. Boxes span upper and lower quartile with median indicated; whiskers represent range. SCAT3 metrics describe **(A)** total number of symptoms reported, and **(B)** summed severity of all symptoms reported.

At baseline prior to the soccer heading, total symptom score and severity were not different between the three conditions (all *p* > 0.80) (Figure 1). There was no significant change in the number or severity of symptoms following sham or control exposures (all *p* > 0.23, Figure 1). By contrast, following soccer heading, there was an increased symptom severity (*p* = 0.03) and a trend toward an elevated number of symptoms (*p* = 0.08) (Figure 1). Five of the seven participants reported a greater number of symptoms and symptom severity following soccer heading, which returned to baseline in all participants at the evening follow-up phone call. The most commonly reported symptoms following soccer heading were headache (71%, 1.3 ± 1.1); pressure in the head (57%, 0.7 ± 0.8); and don't feel right (57%, 0.7 ± 0.8).

### Heart rate variability and baroreceptor sensitivity metrics

**Figure 2:**
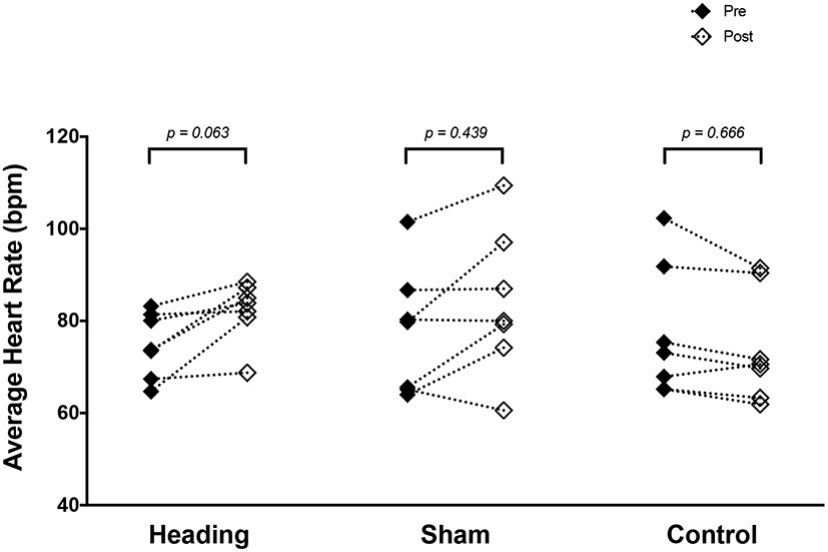
Heart rate measured before and after exposure to heading, sham, and control conditions across all participants. *p*-values are for *a priori* simple effects comparisons.

**Figure 3:**
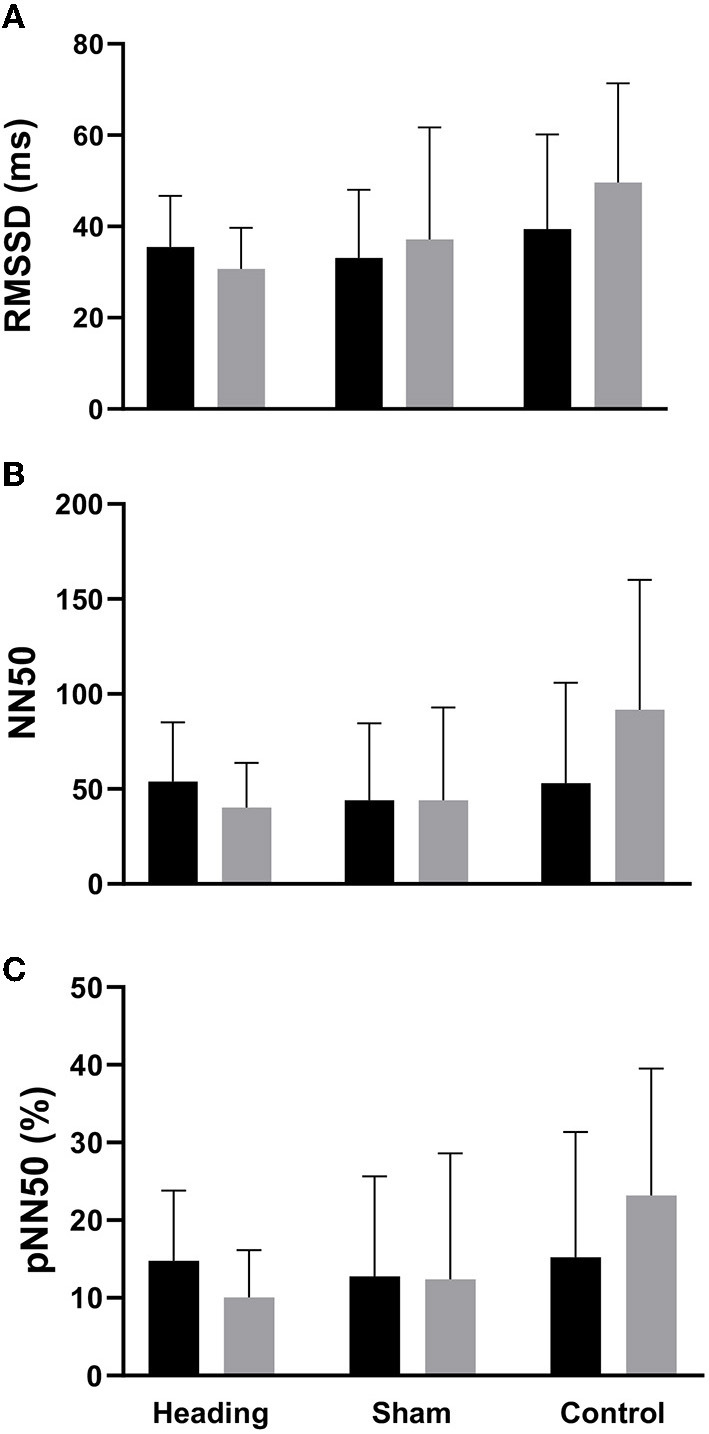
Time-domain HRV metrics before and after exposure to heading, sham, and control conditions across all participants. **(A)** Root-mean square differences of successive R-R intervals (RMSSD), **(B)** Number of adjacent R-R intervals that differ from each other by more than 50 ms (NN50), and **(C)** Percentage of successive R-R intervals that differ by more than 50 ms (pNN50). The columns are average values for the group with the standard deviation being represented with the error bars (*p-*values all >0.05).

**Figure 4:**
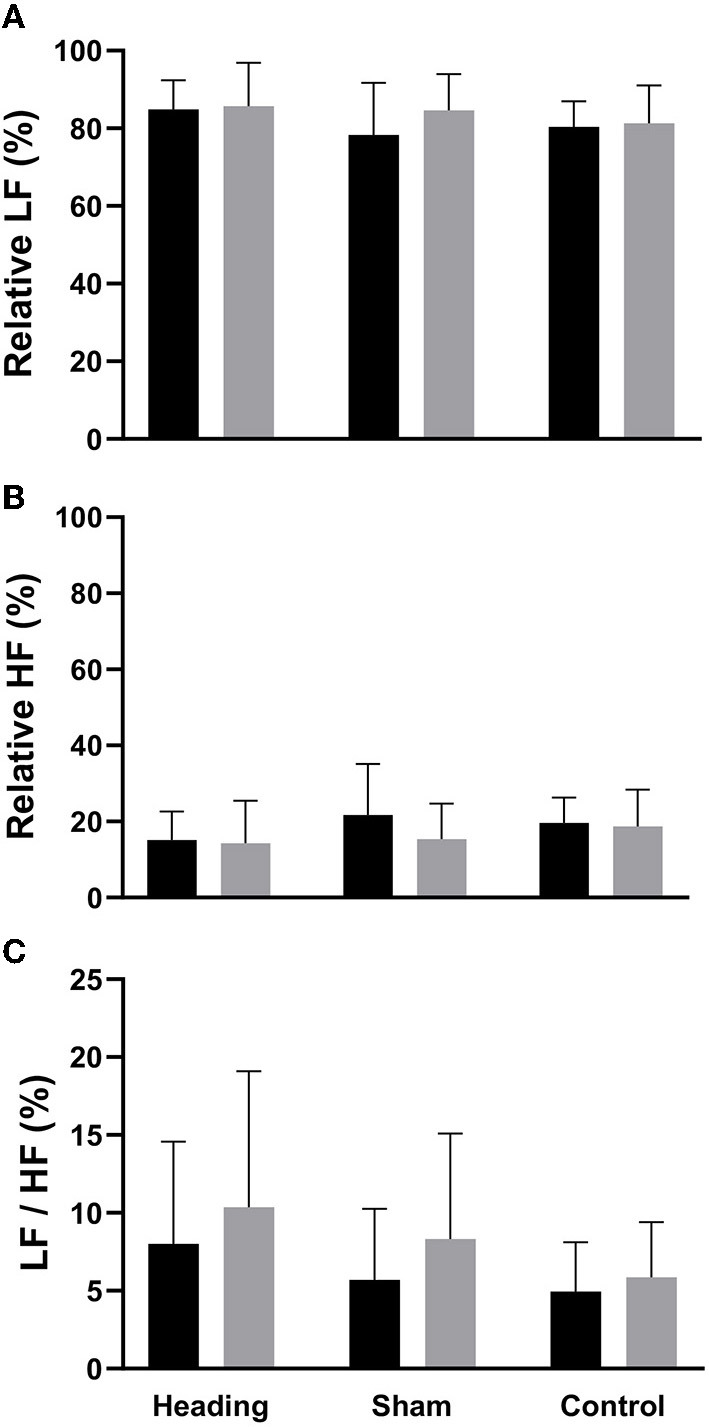
Frequency-domain HRV metrics comparing before and after exposure to heading, sham, and control conditions across all participants. **(A)** relative lower frequency (LF) power. **(B)** relative high frequency (HF) power, and **(C)** LF/HF ratio. The columns are average values for the group with the standard deviation being represented with the error bars (*p-*values all >0.05).

**Figure 5:**
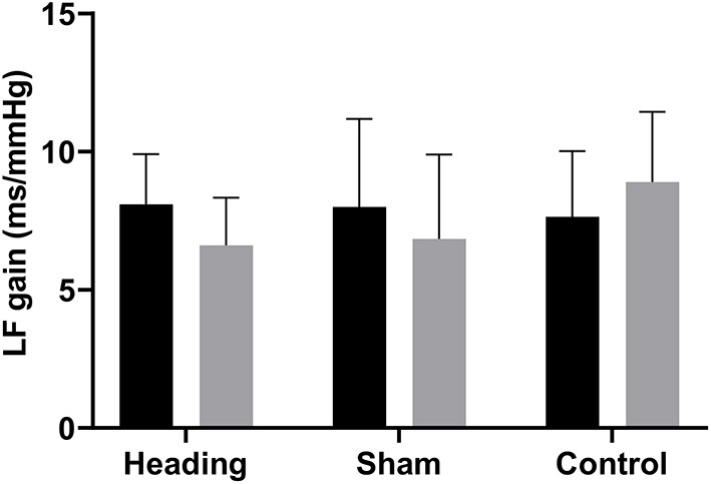
Cardiac baroreceptor sensitivity comparing before and after exposure to heading, sham, and control conditions across all participants, which is represented by the low frequency (LF) gain metrics. The columns are average values for the group with the standard deviation being represented with the error bars (*p-*values all >0.05).

A trend toward an increase in heart rate was noted during the soccer heading condition (*p* = 0.063) when comparing pre (74.8 ± 7.1 bpm) to post (82.3 ± 6.6 bpm) heart rate (Figure 2). Contrarily, there was no significant heart rate change following sham (*p* = 0.439) or control exposures (*p* = 0.666) (Figure 2). Moreover, following all three conditions, no significant change was present in time-domain (all *p* > 0.260) or frequency-domain (all *p* > 0.327: Figures 3, 4) metrics. Congruently, cardiac BRS LF gain was not significantly changed from pre- to post-exposure in all three interventions (all *p* > 0.144) (Figure 5). All of the aforementioned metrics had comparable pre-measures (all *p* > 0.427) between the three interventions (Figures 2–5). Detailed information on the resting physiologic parameters, SAC scores, and HRV and BRS metrics in each condition can be found in Tables 1, 2.

**Table 1:** Resting physiologic parameters and Standardized Assessment of Concussion recorded before and after heading, sham, and control exposures.

**Metric**	**Heading**		**Sham**		**Control**		***p-*** **value**		
	**Pre**	**Post**	**Pre**	**Post**	**Pre**	**Post**	**Condition**	**Time**	**Condition x Time**
MAP (mm Hg)	95.8 (6.2)	91.9 (4.4)	94.2 (6.4)	90.1 (3.2)	91.9 (6.2)	92.6 (5.3)	0.604	0.155	0.439
Respiratory rate (bpm)	15.8 (1.1)	15.6 (1.6)	15.6 (2.4)	15.1 (3.0)	15.0 (4.4)	15.0 (3.9)	0.870	0.646	0.812
IOP (mmHg)	15.6 (3.0)	15.6 (3.0)	14.9 (3.0)	14.9 (3.0)	15.6 (2.7)	15.6 (2.7)	0.807	0.746	0.873
SAC score	26.3 (1.8)	26.3 (1.8)	27.4 (2.6)	27.4 (2.6)	27.9 (1.1)	27.9 (1.1)	0.142	0.719	0.542

Data are presented as mean (SD).

MAP, mean arterial pressure; IOP, intraocular pressure; SAC, Standardized Assessment of Concussion (measure from Sport Concussion Assessment Tool version 3).

**Table 2:** Baroreceptor sensitivity and heartrate variability before and after heading, sham, and control exposures.

**Metric**	**Heading**		**Sham**		**Control**		***p-*** **value**		
	**Pre**	**Post**	**Pre**	**Post**	**Pre**	**Post**	**Condition**	**Time**	**Condition x Time**
BRS (ms/mmHg)	8.1 (1.8)	6.6 (1.7)	8.0 (1.8)	6.8 (3.1)	7.6 (2.4)	8.9 (2.5)	0.566	0.559	0.299
RMSSD (ms)	35.5 (11.2)	30.7 (9.0)	33.1 (14.9)	37.2 (24.6)	39.5 (20.7)	49.7 (21.7)	0.212	0.571	0.547
NN50	53.9 (31.3)	40.3 (23.5)	44.1 (40.5)	44.0 (48.9)	53.1 (52.8)	91.7 (68.3)	0.223	0.568	0.318
pNN50 (%)	14.8 (9.0)	10.1 (6.1)	12.8 (12.9)	12.4 (16.2)	15.2 (16.1)	23.2 (16.2)	0.324	0.817	0.451
LF power (nu)	84.9 (7.5)	85.7 (11.2)	78.3 (13.4)	84.6 (9.3)	80.3 (6.6)	81.3 (9.7)	0.440	0.381	0.707
HF power (nu)	15.1 (7.5)	14.3 (11.2)	21.7 (13.4)	15.4 (9.3)	19.7 (6.6)	18.7 (9.7)	0.440	0.381	0.707

Data are presented as mean (SD).

BRS, baroreceptor sensitivity; RMSSD, root mean square of successive R-R interval differences; NN50, number adjacent NN intervals that differ from each other by more than 50 ms; pNN50, percentage of successive R-R intervals that differ by more than 50 ms; LF power, low frequency power; HF power, high frequency power.

## Discussion

This investigation examined how an acute bout of controlled soccer heading affects autonomic function. The main findings were an acute bout of soccer heading resulted in: (1) an elevation in symptom severity that resolved within 24 h; (2) a trend toward augmented absolute heart rate levels, but no significant change in time- and frequency- HRV domains; and (3) no alterations in cardiac BRS. Taken together, the current data suggest a controlled bout of soccer heading is insufficient to cause short-term alterations in autonomic nervous system function, although it did appear to result in a short-term worsening of symptoms as measured with the SCAT3.

### Comparison to previous research

Several studies have examined the effect acute soccer heading has on various physiological parameters (42–53). This study varies substantially from much of the prior work in that it induced large cumulative linear and rotational impact exposure levels that were beyond those typically observed in match play. We have previously shown in a series of studies that the magnitude of head impact in the current investigation alters blood-based biomarkers associated with concussion (22), neurovascular coupling metrics (21), and cerebral autoregulation metrics (20). Nevertheless, despite these previous findings using the same protocol, there were minimal changes observed with respect to the autonomic nervous system function, aside from a trend toward increased heart rate (Figure 2). The relative lack of agreement between the current findings and those from our previous publications using the same protocol suggests that autonomic nervous system function is less sensitive to the effects of head impact exposure in an acute bout of soccer heading than either cerebrovascular function or blood biomarkers associated with neurological disruption.

The current findings with respect to the autonomic nervous system have some similarities to a study in 2019 by Harriss et al. (19), who noted small and moderate effect size (Cohen's *d*) differences in HRV metrics following acute soccer heading. However, the two studies vary in several key aspects. The current investigation had individuals successfully perform 40 headers with a 20-min span at a distance of ~25 m, whereas Harriss et al. (19), had players engage in just 5 headers, although these were performed in under a minute at a distance of just ~8 m. Moreover, they propelled the ball at 21.6 km/h toward the participants, whereas the current protocol used initial velocity of 77.5 ± 3.7 km/h, resulting in average linear and rotational head accelerations near the upper values previously reported (54). Finally, the previous investigation examined spontaneous HRV and cardiac BRS measures in a supine position; whereas in this study they were taken in an upright position, which is known to reduce variation and enhance reproducibility (55). Given this context, one might have expected larger effects in the current study than in that of Harriss et al. (19). Despite this, both studies found that acute soccer heading has minimal effects on autonomic function.

Furthermore, other reports have noted perturbations in HRV (18, 56, 57) and cardiac BRS (58) metrics following more substantive head impacts (e.g., concussions) among athletes. The exact physiological underpinnings that occur to the autonomic nervous system following concussion are largely unknown; however, this may in part be explained through the metabolic cascade known to occur following concussion (59, 60). This cascade involves diffuse axonal dysfunction and altered neurotransmission. After an initial period of hypermetabolism, there is altered mitochondrial function leading to reduced glucose utilization. Cardiac BRS and HRV are known to be influenced through output from the brain stem and the hypothalamus, respectively (10). Therefore, a cellular or neurometabolic disruption in networks associated with these regions could result in dysautonomia which may partially explain these deficits seen more often following concussion, as opposed to the controlled soccer-heading performed in the current investigation. Future research is needed to understand the total exposure required to cause immediate changes in different aspects of brain function.

Whether and how such alterations are related to the development of longer-term neurodegenerative disorders observed in former soccer players (6–9) is currently unknown. The results from this study suggest changes in autonomic function as probed by HRV are unlikely to be involved at least as they relate to sub-concussive soccer heading impacts. Clearly, other pathophysiological processes and/or exposure to concussive impacts must play a role in these longer-term clinical outcomes.

### Limitations

A prominent limitation of the current study is the small sample size, which is likely linked to the non-significant findings. Nonetheless, a pseudo-random crossover design was used to reduce the likelihood any covariates (i.e., concussion history, genetics, soccer experience, etc.) (61, 62) influenced the findings, and also optimized statistical power while exposing fewer participants to potentially hazardous conditions. Therefore, we believe the results provide pertinent knowledge to the literature, given the cumulative impact of heading exposure and the comparable sample size to previous soccer heading studies. Additionally, the group recruited were exclusively male soccer players which is important as female soccer players have one of the highest rates of concussion among female athletes (63). However, despite methodological differences, these results are similar to a published study examining HRV in female adolescents (19). Furthermore, we also had no way of blinding participants to their exposure (heading, sham, or control), which could induce alterations to sympathetic and parasympathetic tone, thereby changing absolute heart rate and potentially HRV metrics. The SCAT3 has recently been shown to have only moderate reliability and specificity in diagnosing concussion and post-concussive symptoms (64) and, thus, its utility in the context of this study can be questioned. Moreover, as the SCAT3 relies on subjective self-report, participants may have been more likely to report symptoms knowing they had just been exposed to a controlled bout of repeated head impacts. Nonetheless, as the methodology in this study used objective outcomes [short-term HRV/cardiac BRS: (23, 24, 37–39)] and validated questionnaires (36) the risk of bias from this is minimal. Finally, skin-worn impact sensors can potentially overestimate head-impact exposure levels as a result of skin-based movement artifact during impacts (65–67). It is possible this may have occurred during the current investigation and the absolute values reported in this manuscript may have overestimated the exposure levels. Despite all these, the present findings are important given the lack of autonomic data presented within the current literature on soccer heading.

## Conclusion

This investigation found exposure to 40 soccer head impacts elevates symptom severity and a trend toward an increase in absolute heart rate, although no alterations were found in HRV and cardiac BRS. These findings add to the evolving body of literature regarding the alterations in brain structure and function resulting from an acute bout of soccer heading. Future experiments are needed to comprehensively understand acute changes that occur to the autonomic nervous system and the associated structures across the lifetime of heading in a soccer career. Finally, investigations into the impact of soccer heading on HRV and cardiac BRS response changes with respect to age, sex, soccer skill level, and accumulative exposure are also warranted.

## Data availability statement

The raw data supporting the conclusions of this article will be made available by the authors, without undue reservation.

## Ethics statement

The studies involving human participants were reviewed and approved by University of British Columbia Clinical Research Ethics Board (H14-00368). The patients/participants provided their written informed consent to participate in this study.

## Author contributions

JS, AW, and PvD designed the study. JS, JB, AW, KB, and JD collected data. All authors contributed to data interpretation and writing and/or editing the manuscript.

## Funding

This research was funded by grants from CIHR (183304) and CFI (30979) awarded to PvD. AW was supported by the Vanier Canada Graduate Scholarships program, the O'Brien Foundation Fellowship, and the Vancouver Coastal Health-CIHR-UBC MD/PhD Studentship. JS was supported by the Innovations in Wellness post-doctoral fellowship.

## Conflict of interest

The authors declare that the research was conducted in the absence of any commercial or financial relationships that could be construed as a potential conflict of interest.

## Publisher's note

All claims expressed in this article are solely those of the authors and do not necessarily represent those of their affiliated organizations, or those of the publisher, the editors and the reviewers. Any product that may be evaluated in this article, or claim that may be made by its manufacturer, is not guaranteed or endorsed by the publisher.
